# Sex-Specific Association of Rasagiline with Motor Progression in *GBA1*-Associated Parkinson’s Disease

**DOI:** 10.3390/life16071103

**Published:** 2026-07-01

**Authors:** Hodaya Saragani, Rebecca Henner, Michal Becker-Cohen, Shoshana Revel-Vilk, Ari Zimran, Iris Harari, Roni Eichel, Gilad Yahalom, Mikhal E. Cohen

**Affiliations:** 1 Faculty of Medicine, Hebrew University of Jerusalem, Jerusalem 9112102, Israel; svilk@szmc.org.il (S.R.-V.); azimran@gmail.com (A.Z.); eichel@szmc.org.il (R.E.); gilady@szmc.org.il (G.Y.); mikhalc@szmc.org.il (M.E.C.); 2 Stern College for Women, Yeshiva University, 500 W 185th St, New York, NY 10033, USA; rhenner@mail.yu.edu; 3 Gaucher Unit, The Eisenberg R&D Authority, Shaare Zedek Medical Center, Jerusalem 9103102, Israel; michalbc@szmc.org.il; 4 Agyany Pharma Ltd., 14 HaDishon, Jerusalem 9695614, Israel; 5 Assuta Ashdod Medical Center, 7 HaRefuah Way, Ashdod 7747629, Israel; irismajere@gmail.com; 6 Movement Disorders Unit and The Eisenberg R&D Authority, Shaare Zedek Medical Center, Jerusalem 9103102, Israel; 7 Department of Neurology and The Eisenberg R&D Authority, Shaare Zedek Medical Center, Jerusalem 9103102, Israel

**Keywords:** Parkinson’s disease, rasagiline, *GBA1*, biological sex, disease progression

## Abstract

Variants in the glucocerebrosidase gene (*GBA1*) are the predominant genetic risk factor for Parkinson’s disease (PD), often accelerating disease progression. While biological sex modulates PD progression, the longitudinal association between rasagiline (a monoamine oxidase-B [MAO-B] inhibitor) and motor outcomes in *GBA1*-associated Parkinson’s disease (*GBA1*-PD) remains unclear. This retrospective cohort study (2019–2026) analyzed 259 patients with PD (106 females, 153 males) with a median follow-up of 1.56 years to evaluate the association between rasagiline use and motor decline, emphasizing sex-stratified outcomes. Motor progression was evaluated using the Movement Disorder Society—Unified Parkinson’s Disease Rating Scale part III (MDS-UPDRS-III). Rates of change were calculated using sex-stratified Generalized Estimating Equations models, with adjustment for age at diagnosis to evaluate treatment effects and sex-specific associations. Among 259 patients, rasagiline use was associated with a significantly slower annual rate of motor decline (Slope Difference: −0.95; *p* = 0.03). In the *GBA1*-PD subgroup, females using rasagiline exhibited a clinically relevant slower rate of progression (approximately 1 point/year on the MDS-UPDRS-III) compared with non-users, although not statistically significant (*p* = 0.08); no association was observed in males. These findings suggest a potential sex-specific association of rasagiline with motor progression in *GBA1*-PD. Results highlight the importance of sex-stratified analyses to support personalized therapeutic approaches for PD genetic variants.

## 1. Introduction

The glucocerebrosidase type I gene (*GBA1*) encodes the lysosomal enzyme glucocerebrosidase, which is involved in the metabolism of sphingolipids. Homozygous variants in the *GBA1* gene cause Gaucher disease, and heterozygous variants are a major risk factor for *GBA1*-related Parkinson’s disease (*GBA1*-PD), with odds ratios ranging from 2.2 to 30 [1]. *GBA1* variants accelerate motor and cognitive decline through endoplasmic reticulum stress, impaired autophagy–lysosomal pathways, and altered lipid homeostasis, which facilitate alpha-synuclein accumulation [1]. Consequently, patients with *GBA1*-PD (Sidransky syndrome) typically exhibit more rapid motor impairment, as measured by the Movement Disorders Society—Unified Parkinson’s Disease Rating Scale part III (MDS-UPDRS-III), and a markedly increased risk of early dementia (hazard ratio >5) [2,3]. Alternatively, a subset of patients with *GBA1*-PD follow an exceptionally mild clinical course, the determinants of which remain unelucidated [4]. This phenotypic heterogeneity suggests that biological sex or therapeutic associations may modulate the clinical trajectory in *GBA1*-PD.

Sex differences in *GBA1*-PD are nuanced and variant-specific. Although *GBA1*-PD generally shows a male predominance, carriers of severe *GBA1* variants (e.g., L444P) are more often female, whereas carriers of mild variants (e.g., N370S) are more often male [5]. Furthermore, while idiopathic PD typically develops earlier in men than in women (mean 57.6 vs. 59.3 years), this pattern is reversed in *GBA1*-PD, where female carriers exhibit a significantly younger age of onset than males (mean 53.2 vs. 55.3 years) [6]. Currently, there is no robust evidence for sex-based differences in motor or cognitive decline rates among *GBA1*-PD patients.

Beyond clinical phenotyping, biological sex modulates the neurobiological environment in PD. Males exhibit a more pronounced oxidative stress signature and lower antioxidant capacity than females, who appear to possess more resilient cellular defense mechanisms [7,8]. Furthermore, while males show greater vulnerability to nigrostriatal dysfunction, females often demonstrate more effective compensatory adaptation and higher dopamine transporter binding [9]. These innate biological variations suggest that the pathophysiological substrate of *GBA1*-driven neurodegeneration may differ by sex.

The monoamine oxidase B (MAO-B) enzyme degrades dopamine and other biogenic amines. Rasagiline and selegiline are irreversible MAO-B inhibitors and have demonstrated several neuroprotective properties in cellular and animal models, including mitochondrial preservation, alpha-synuclein mitigation, and upregulation of antioxidant enzymes such as superoxide dismutase and glutathione [10,11]. While selegiline showed symptomatic benefit and delayed the need for levodopa, it did not conclusively demonstrate disease modification [12]. Clinical trials using delayed-start designs, such as the ADAGIO study, suggest a possible disease-modifying effect for rasagiline, yet results were inconsistent across doses and relied on subgroup analyses [13]. This lack of reproducibility underscores the need for a more personalized approach, focusing on specific genetic and biological factors that may modulate the therapeutic association of MAO-B inhibition.

The therapeutic role of MAO-B inhibitors in *GBA1*-PD is poorly characterized in the literature. While clinical phenotypes and pharmacological patterns in *GBA1*-PD appear similar to those in idiopathic PD [14], mechanistic studies suggest that reduced glucocerebrosidase activity resulting from *GBA1* variants may influence neurotransmitter metabolism by increasing MAO-B activity and dopamine turnover [15]. Specifically, impaired mitochondrial function in *GBA1* variant carriers can lead to cytosolic dopamine accumulation, which is metabolized by MAO-B into toxic reactive oxygen species [16].

Despite observations that *GBA1*-PD patients respond to dopaminergic therapies similarly to idiopathic PD, the disease-modifying potential of MAO-B inhibitors remains a critical area of investigation, particularly in high-risk genetic subgroups. Given the accelerated neurodegeneration associated with *GBA1*-PD and the emerging evidence of biological sex as a modulator of PD progression, evaluating whether the therapeutic association of MAO-B inhibition is modified by sex in *GBA1* variant carriers is essential.

The present study evaluates the longitudinal association between rasagiline use and motor progression in a cohort of patients with *GBA1*-PD. To address the potential influence of biological sex on disease trajectory and treatment response, we utilized sex-stratified analyses. This research examines whether specific subpopulations exhibit distinct clinical patterns associated with rasagiline treatment, aiming to provide evidence for personalized therapeutic strategies for *GBA1*-PD.

## 2. Materials and Methods

### 2.1. Study Design and Participants

This retrospective cohort study was conducted and reported in accordance with the Strengthening the Reporting of Observational Studies in Epidemiology (STROBE) reporting guideline [17]. The study is a longitudinal analysis of clinical data from the Shaare Zedek Medical Center PD cohort, collected between 2019 and 2026. We included consecutive patients who attended the Movement Disorders Unit with a clinical diagnosis of PD, established by movement disorder specialists (GY, MEC) in accordance with Movement Disorder Society clinical criteria [18], and who agreed to undergo genetic testing for PD-associated variants. Each sample underwent next-generation sequencing using a PD-associated gene panel as (CENTOGENE GmbH, Rostock, Germany), previously mentioned [19].

A total of 259 consecutive patients with PD were included in the final longitudinal analysis, comprising 106 females (40.9%; median age at diagnosis: 66.0 years [IQR: 57.2–72.0]) and 153 males (59.1%; median age at diagnosis: 62.0 years [IQR: 54.0–70.0]). Within this study population, a genetically defined subgroup of 64 patients with *GBA1*-PD was analyzed, including 29 females (median age at diagnosis: 58.0 years [IQR: 52.0–66.0]) and 35 males (median age at diagnosis: 56.0 years [IQR: 53.0–61.0]). Detailed baseline clinical, motor severity (MDS-UPDRS-III), and demographic characteristics of the total cohort and the genetic subgroups are explicitly summarized in Table 1.

**Figure 1:**
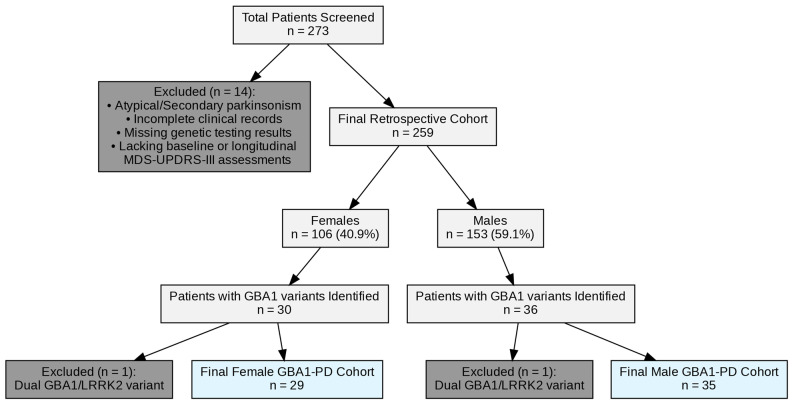
Patient screening, exclusion criteria, and cohort stratification by sex and *GBA1* variant status. Flow diagram illustrating the selection process for the retrospective cohort (*n* = 259). *GBA1* indicates the glucocerebrosidase gene; *GBA1*-PD, *GBA1*-associated Parkinson’s disease; PD, Parkinson’s disease.

To ensure cohort homogeneity and methodological transparency, strict exclusion criteria were applied. We excluded patients with atypical parkinsonism (e.g., progressive supranuclear palsy, multiple system atrophy, or corticobasal degeneration) or secondary parkinsonism (e.g., vascular, toxic, or drug-induced). Additionally, patients with incomplete clinical records, missing genetic testing results, or those completely lacking baseline or subsequent longitudinal Movement Disorder Society—Unified Parkinson’s Disease Rating Scale part III (MDS-UPDRS-III) assessments were excluded from the analysis. Consistent with our statistical framework designed to handle varying follow-up lengths, insufficient follow-up duration was not used as a reason for exclusion to minimize recruitment and selection bias. The detailed step-by-step patient selection, exclusion process, and subsequent cohort stratification are visually outlined in Figure 1.

### 2.2. Ethical Approval and Informed Consent

This study was conducted in accordance with the Declaration of Helsinki and was approved by the Institutional Review Board of Shaare Zedek Medical Center, Jerusalem, Israel (Helsinki Committee; approvals 0028-20 and 0377-21). Participants provided written informed consent for genetic testing and data collection at enrollment (0028-20). For the retrospective analysis of de-identified clinical data (0377-21), the board granted a waiver of informed consent in accordance with institutional policy. The authors ensure complete anonymity of all participants in accordance with the journal requirements.

### 2.3. Clinical Assessment

Clinical severity and disease progression were assessed using the MDS-UPDRS-III (Motor Examination) score [18]. All assessments were performed by movement disorder specialists at our center using a standardized protocol to ensure consistency. Clinical assessments were predominantly performed during the patients’ ‘ON’ state, as part of routine clinical follow-up. While a small minority of assessments may have occurred in the ‘OFF’ state due to the retrospective nature of the study, most of the data reflect the patients’ typical functional status under their regular dopaminergic treatment. To maximize statistical power and avoid selection bias, no minimum follow-up duration was mandated; instead, a Generalized Estimating Equations (GEE) framework was utilized to account for varying follow-up lengths across the cohort. For longitudinal analyses, patients were stratified by sex to evaluate differential therapeutic associations.

Rasagiline use was defined as the administration of a standardized dose of 1 mg/day as part of the patient’s clinical management. Notably, rasagiline was the sole MAO-B inhibitor utilized in this cohort. Patients were included regardless of whether rasagiline was administered as monotherapy or as an adjunct to other dopaminergic treatments (e.g., levodopa or dopamine agonists). Given the uniform clinical dosing across the cohort, dose–response analyses were not performed, and treatment was analyzed as a binary longitudinal covariate (user vs. non-user).

### 2.4. Statistical Analysis

Descriptive statistics were used to summarize baseline characteristics. Continuous variables were assessed for normality using the Shapiro–Wilk test; as data exhibited non-normal distributions, they are presented as median [interquartile range] and compared using the Mann–Whitney U test (selected as a non-parametric alternative justified by the non-normal distribution of continuous variables). Categorical variables are presented as numbers (percentages) and compared using the Chi-square or Fisher’s exact test.

For the primary longitudinal analysis, we employed Generalized Estimating Equations models. This approach was selected because it accounts for within-subject correlations in repeated measurements while effectively handling unbalanced data, including patients with varying numbers of follow-up visits without requiring a minimum follow-up duration, thereby minimizing selection bias. The models utilized a Gaussian family with an exchangeable correlation structure to model within-subject dependencies.

The primary outcome was the rate of change in MDS-UPDRS-III scores per year, evaluated via the interaction term between time (years since diagnosis) and rasagiline use (Time × rasagiline). Rasagiline use was treated as a time-varying covariate in the Generalized Estimating Equations models, incorporating clinical data from participants who initiated therapy during follow-up. To ensure clinical relevance, rasagiline status was determined from documented, stable therapeutic use in medical records rather than from transient exposure.

To address potential confounding and baseline imbalances in medication use between sexes and to explore biological sex as a known moderator of disease progression, a pre-specified stratified analysis by sex was performed to estimate sex-specific clinical patterns. All models were adjusted for age at diagnosis as a continuous covariate to control for its established influence on motor decline.

Statistical analyses were conducted using Python (v3.12.13; Python Software Foundation) with the Pandas (v2.2.2) and Statsmodels (v0.14.6) libraries (https://colab.research.google.com/ accessed on 29 June 2026). All tests were two-tailed, and a *p* value of <0.05 was considered statistically significant. The statistical analytic code utilized for these analyses is available upon reasonable request to the corresponding author.

### 2.5. Artificial Intelligence Statement

Generative artificial intelligence (Gemini 3 Flash, Google) was used solely for linguistic editing and technical support during the development of the statistical code. The authors reviewed and verified all AI-generated content to ensure accuracy and take full responsibility for the manuscript’s integrity.

## 3. Results

### 3.1. Study Population and Baseline Characteristics

Of 273 patients screened, 14 were excluded due to insufficient clinical parameters: 12 lacked any motor assessments (MDS-UPDRS-III scores or exam dates), and 2 had missing core demographic data required for the longitudinal models. Consequently, a final retrospective cohort of 259 patients with PD (106 females [40.9%] and 153 males [59.1%]) was included in the analysis.

For the genetic sub-analysis, we focused on 64 patients with *GBA1*-PD (29 females and 35 males), after excluding 2 subjects (3.1%) with dual *GBA1* and Leucine-rich repeat kinase 2 (*LRRK2*) variants to ensure a more homogeneous genetic phenotype. Within the *GBA1* cohort, the genotype distribution included 52 (81.2%) heterozygotes and 8 (12.5%) homozygotes or compound heterozygotes (Gaucher disease). The study flow and cohort stratification are summarized in Figure 1.

Baseline demographic and clinical characteristics for the total cohort (*n* = 259) and the *GBA1*-PD subgroup (*n* = 64), stratified by sex, are presented in Table 1.

In the total cohort, no significant sex differences were observed at baseline regarding age at diagnosis, motor severity (MDS-UPDRS-III score), the proportion of rasagiline ever-users, or the distribution of individuals with *GBA1*-PD (all *p* ≥ 0.08). Within the *GBA1*-PD subgroup, baseline characteristics were well-balanced between sexes, with no statistically significant differences in age at diagnosis or baseline motor severity. Regarding concomitant therapies, although only one female received Ambroxol, a higher proportion of males received rasagiline at baseline compared to females (60.0% vs. 27.6%; *p* = 0.01). Other baseline clinical and demographic characteristics were largely comparable.

### 3.2. Longitudinal Motor Progression in the Total Cohort

First, we evaluated the longitudinal association between rasagiline use and motor progression. The longitudinal Generalized Estimating Equations analysis included 259 patients, yielding 948 observations (mean 3.66 per patient). In this model, all 259 patients contributed to estimating the baseline clinical state (intercept), while the annual rate of progression (slope) was estimated from 176 patients (68.0%) with two or more assessments. These longitudinal contributors provided a median follow-up of 1.56 years (range: 0.15–5.4 years).

Using Generalized Estimating Equations models, higher age at diagnosis was significantly associated with overall motor severity (Coefficient: 0.24; *p* < 0.001), reflecting a higher baseline intercept for those diagnosed at an older age. In the total study population, use of rasagiline at any time during follow-up was independently associated with a slower annual rate of motor progression, with an annual slope difference of −0.95 points (*p* = 0.03; Table 2). This represents an attenuation of nearly 1.00 point per year in the MDS-UPDRS-III compared with never users. However, when testing for a sex-specific association, the triple interaction between sex, time, and rasagiline use was not statistically significant (*p* = 0.41), indicating that the association between rasagiline use and motor progression was similar in males and females across the cohort.

### 3.3. Longitudinal Motor Progression in Patients with GBA1-PD

Within the *GBA1*-PD subgroup (*n* = 64), we employed Generalized Estimating Equations models adjusted for age at diagnosis to evaluate the longitudinal association between rasagiline use and MDS-UPDRS-III scores. In this model, all 64 patients contributed to estimating the baseline clinical state (intercept), while the annual rate of progression (slope) was estimated from 47 patients (73.4%) with two or more assessments. These longitudinal contributors provided an average of 4.3 observations per patient, with a median follow-up of 1.1 years (range: 0.15–5.3 years).

**Figure 2:**
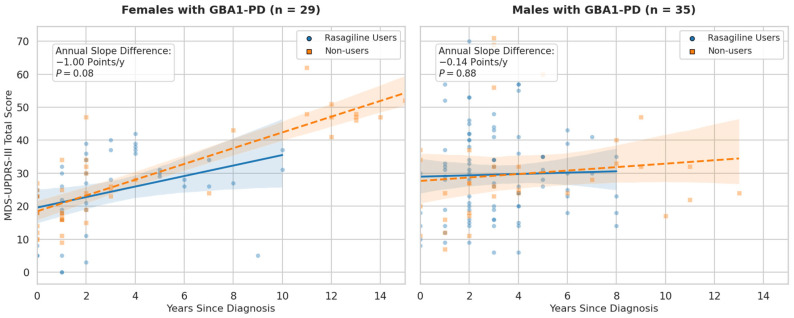
Longitudinal association between rasagiline use and motor progression in patients with *GBA1*-PD stratified by sex. Longitudinal motor progression in female (**left**) and male (**right**) patients with *GBA1*-PD. Lines represent the estimated annual rate of change in MDS-UPDRS-III scores for rasagiline users (solid blue line, circles; 1 mg/day) versus non-users (dashed orange line, squares). Shaded areas indicate 95% confidence intervals. MDS-UPDRS-III indicates the Movement Disorder Society—Unified Parkinson’s Disease Rating Scale Part III.

Among females with *GBA1*-PD (*n* = 29), those who used rasagiline at any point during the study (ever-users, *n* = 11) demonstrated a slower rate of motor progression compared with never-users (*n* = 18). Specifically, rasagiline ever-use was associated with an estimated annual attenuation of 1.00 point in the total MDS-UPDRS-III score (Annual Slope Difference: −1.00 points/year; *p* = 0.08; Table 3). However, this difference did not reach statistical significance (Figure 2).

Conversely, no such association was observed among males with *GBA1*-PD (*n* = 35), where a higher prevalence of rasagiline use was reported (ever-users, *n* = 25). In this subgroup, patients exhibited nearly identical rates of annual motor progression irrespective of their treatment status, demonstrating no clinical or statistical benefit for rasagiline use in males (Annual Slope Difference: −0.14; *p* = 0.88).

## 4. Discussion

In this longitudinal cohort study, rasagiline use was associated with a statistically significant slower annual rate of motor progression in the overall PD cohort, reflecting an estimated attenuation of approximately one point per year in the expected increase in the MDS-UPDRS-III score. This association was consistently observed across both sexes over an extensive longitudinal follow-up period. However, within the *GBA*1-PD subgroup, a numerical difference was observed specifically among females. In this group, rasagiline use was associated with a slower motor decline of one point per year; although this difference did not reach statistical significance, its magnitude was clinically comparable to the association observed in the total cohort. No such association was observed in males with *GBA1*-PD.

While MAO-B inhibitors are widely utilized for their symptomatic benefits and their capacity to delay levodopa initiation, their long-term disease-modifying potential remains a subject of debate [12]. Unlike levodopa, which remains the symptomatic gold standard but is limited by long-term motor complications like dyskinesias, rasagiline offers a more favorable complication profile [13] alongside preclinical neuroprotective properties, such as mitochondrial preservation [10,11]. Given that *GBA1* variants aggressively disrupt lysosomal-mitochondrial homeostasis, these features may offer distinct pathophysiological advantages in this genetic subgroup. Our findings suggest that, while the association between rasagiline use and slower motor progression is evident in the general PD population, its strength may vary with the interplay between biological sex and *GBA1* variants. This could explain why previous studies that did not account for these factors reported inconsistent results.

The association in females was observed alongside a higher frequency of rasagiline use among males at study entry. By utilizing sex-stratified models, we evaluated the association within each sex independently. This approach confirmed that the slower progression observed in females was independent of treatment frequency. Furthermore, the lack of baseline motor differences between sexes suggests that this divergence reflects a sex-specific association rather than initial phenotypic variation.

Several potential pathways may explain this dimorphism, addressing a critical gap in the existing literature regarding how therapeutic responses are modulated in genetically defined PD subgroups. Historically, large clinical trials evaluating the disease-modifying potential of MAO-B inhibitors, such as the landmark ADAGIO study, yielded inconsistent and highly debated results [13]. A major factor contributing to this lack of reproducibility is that prior investigations routinely evaluated heterogeneous patient populations as a single entity, without accounting for the distinct interplay between specific genetic variants and biological sex. Emerging evidence suggests that biological sex and *GBA1* mutations interact synergistically to influence the clinical phenotype and the underlying neurodegenerative cascade. Functionally, *GBA1* variants aggressively disrupt lysosomal glucocerebrosidase activity, precipitating impaired autophagy–lysosomal pathways and accelerated alpha-synuclein accumulation [1]. This lysosomal dysfunction leads to a downstream failure in mitochondrial function, causing severe oxidative stress through the accumulation of toxic reactive oxygen species [15,16]. To counteract this cascade, females appear to possess more resilient baseline cellular defense mechanisms and higher antioxidant capacity, with relatively preserved glutathione pathways compared with males [7,8]. Consequently, the secondary antioxidant properties of MAO-B inhibitors likely synergize more effectively within this female biological environment, which is strongly supported by preclinical evidence demonstrating that estrogen (17β-estradiol) directly stimulates the expression of superoxide dismutase (SOD1 and SOD2) and upregulates catalase, thereby reducing toxic free radicals in the substantia nigra [20].

Furthermore, this hormonal priming may interface uniquely with the neuroprotective profile of rasagiline. Beyond its primary role in stabilizing synaptic dopamine, rasagiline’s specific chemical structure exerts independent anti-apoptotic and neuroprotective effects by maintaining mitochondrial membrane potential and preserving complex I activity [10,11]. In female carriers, this therapeutic stabilization can reinforce an already resilient cellular environment, effectively counteracting the metabolic stress induced by accelerated dopamine turnover and cytosolic accumulation of reactive oxygen species characteristic of *GBA1* deficiency [15,16]. Conversely, in males, who exhibit more pronounced baseline oxidative stress levels [7,8] and lack this protective hormonal synergy, the standard clinical dose of rasagiline may be insufficient to halt the aggressive, *GBA1*-driven pathological cascade. Additionally, females demonstrate more effective dopaminergic compensatory adaptation and higher dopamine transporter binding in the nigrostriatal system [9], which may be optimally sustained by the continuous synaptic dopamine availability provided by steady MAO-B inhibition.

These findings suggest potential clinical implications for precision medicine in PD, highlighting how the interaction between biological sex and genetic variants may influence therapeutic associations. For females with *GBA1*-PD, early initiation of rasagiline might offer a strategic advantage, although further prospective studies are warranted to confirm this observation. Ultimately, our results reinforce the necessity for sex-stratified analyses in clinical trials to avoid masking such subgroup-specific associations.

## 5. Strengths and Limitations

Strengths of this study include a longitudinal design with frequent clinical observations and the incorporation of rasagiline use as a time-varying covariate. This statistical approach maximized the use of longitudinal data and captured real-world therapeutic dynamics, including treatment initiation during follow-up. Furthermore, the use of sex-stratified Generalized Estimating Equations models enabled a clear evaluation of associations within each sex independently, effectively accounting for baseline imbalance in rasagiline use frequency. The well-balanced age and baseline motor severity within the *GBA1*-PD subgroups further support these longitudinal findings.

Study limitations must be acknowledged. First, the *GBA1*-PD cohort may be subject to recruitment bias; our center serves as a referral site for an ongoing clinical trial (Ambroxol) enrolling patients from multiple referring centers. Consequently, our cohort includes participants who are younger and have shorter disease duration, per the trial’s inclusion criteria. While this recruitment process influences the cohort’s demographic profile, data consistency was maintained as all clinical assessments were performed locally by our movement disorder specialists using a standardized protocol. Second, as a retrospective analysis of clinical records, standardized timing of assessments relative to medication intake was not strictly mandated. However, most patients were assessed in the ‘ON’ state during routine clinic visits. Any potential variability introduced by the clinical state (ON vs. OFF) is expected to be minimal and was partially addressed by the Generalized Estimating Equations framework, which accounts for intra-subject correlations. Third, this was a single-center, retrospective study evaluating a single MAO-B inhibitor (rasagiline), limiting control over all variables and leading to missing data. Third, the limited number of females with *GBA1*-PD (*n* = 29) likely explains why the findings showed a numerical difference (*p* = 0.08) rather than formal statistical significance; however, the magnitude of the clinical observation (approximately one point annually) remains highly relevant. Finally, the timing and duration of rasagiline initiation were not standardized.

## 6. Conclusions

This longitudinal study provides further clinical evidence of a therapeutic association between rasagiline use and slower motor progression in patients with PD. Our findings suggest that this association is evident in the general PD population and remains clinically meaningful within specific subgroups, such as females with *GBA1*-PD. The ongoing debate regarding the disease-modifying potential of MAO-B inhibitors may be partly explained by the complex interplay between genetic status and biological sex. These results underscore the importance of individualized treatment approaches and highlight the need for sex-stratified analyses in future clinical trials of PD.

## Figures and Tables

**Table 1:** Baseline characteristics of the total study cohort and patients with *GBA1*-PD stratified by sex.

Variable ^a^	Total Cohort (*n* = 259)			Patients with *GBA1*-PD (*n* = 64) ^e^		
	Females (*n* = 106)	Males (*n* = 153)	*p* Value	Females (*n* = 29)	Males (*n* = 35)	*p* Value
Age at Diagnosis, median [IQR], years	66.0 [57.2–72.0]	62.0 [54.0–70.0]	0.08 ^b^	58.0 [52.0–66.0]	56.0 [53.0–61.0]	0.37 ^b^
Baseline MDS-UPDRS-III, median [IQR] ^d^	25.0 [18.0–31.8]	27.0 [20.0–37.0]	0.08 ^b^	23.0 [17.0–28.0]	25.0 [15.5–33.0]	0.51 ^b^
Rasagiline Users, *n* (%)	39/106 (36.8)	60/153 (39.2)	0.79 ^c^	8/29 (27.6)	21/35 (60.0)	**0.01** ^c^
Patients with *GBA1* variants (excluding *LRRK2* dual carriers), *n* (%) ^e^	29/106 (27.4)	35/153 (22.9)	0.50 ^c^	NA	NA	NA
Ambroxol Users, *n* (%)	NA	NA	NA	1/29 (3.4)	0/35 (0.0)	0.45 ^c^
Gaucher disease (2 alleles), *n* (%)	NA	NA	NA	2/29 (6.9)	6/35 (17.1)	0.28 ^c^

Abbreviations: *GBA1*, glucocerebrosidase gene; IQR, interquartile range; *LRRK2*, Leucine-rich repeat kinase 2; MDS-UPDRS-III, Movement Disorder Society—Unified Parkinson’s Disease Rating Scale Part III; NA, not applicable; PD, Parkinson’s disease. ^a^ Data are presented as median [IQR] for continuous variables and as number (percentage) for categorical variables. ^b^ *p* values for continuous variables were calculated using the Mann–Whitney U test. ^c^ *p* values for categorical variables were calculated using the Chi-square test or Fisher’s exact test where appropriate. ^d^ Baseline MDS-UPDRS-III total score refers to the motor severity score at the first documented visit during the study period. ^e^ The *GBA1*-PD group includes only individuals with *GBA1* variants and excludes two participants (one female, one male) who were dual carriers of both *GBA1* and *LRRK2* variants to ensure consistency with subgroup and longitudinal analyses. Bold values indicate statistical significance (*p* < 0.05).

**Table 2:** Longitudinal association between rasagiline use and motor progression in the total cohort (*n* = 259).

Variable	Coefficient (SE) ^a^	*p* Value ^b^	95% CI
Intercept	6.30 (4.52)	0.16	[−2.56, 15.17]
Age at Diagnosis	0.24 (0.06)	**<0.001**	[0.11, 0.36]
Time Since Diagnosis (Years)	1.31 (0.28)	**<0.001**	[0.75, 1.85]
Time × Rasagiline Interaction ^c^	−0.95 (0.44)	**0.03**	[−1.81, −0.08]
Sex (Male)	6.23 (1.87)	**0.001**	[2.55, 9.90]
Time × Sex Interaction ^d^	−0.39 (0.35)	0.27	[−1.07, 0.30]
Rasagiline × Sex Interaction ^e^	−5.18 (3.60)	0.15	[−12.24, 1.89]
Time × Rasagiline × Sex Interaction ^f^	0.44 (0.53)	0.41	[−0.61, 1.49]

Abbreviations: CI, confidence interval; MDS-UPDRS-III, Movement Disorder Society—Unified Parkinson’s Disease Rating Scale Part III; SE, standard error. ^a^ Coefficients represent the estimated mean annual change in MDS-UPDRS-III points; a positive value indicates motor progression, and the null hypothesis value is 0. ^b^ *p* values for interaction terms represent the statistical significance of the difference in clinical patterns between the specified subgroups. ^c^ The Time × rasagiline interaction term represents the difference in the annual rate of motor score change (slope) between rasagiline users and non-users across the general cohort. ^d^ The Time × Sex interaction term evaluates whether the annual rate of motor score change (disease progression) differs significantly between males and females, independent of medication use. ^e^ The rasagiline × Sex interaction term assesses baseline differences in motor severity associated with rasagiline use between sexes at study entry. ^f^ The Time × rasagiline × Sex triple interaction term evaluates whether the clinical association between rasagiline use and the annual rate of motor progression is significantly modified by biological sex. Bold values indicate statistical significance (*p* < 0.05).

**Table 3:** Longitudinal association between rasagiline use and motor progression in patients with *GBA1*-PD stratified by sex.

Sex	Rasagiline Ever-Users, *n*	Total Observations (Mean per Patient)	Annual Slope Difference (Points/Year) ^a,b^	*p* Value ^c^	%95 CI
Females (*n* = 29)	11	95 (3.28)	−1.00	0.08	[−2.11, 0.11]
Males (*n* = 35)	25	124 (3.54)	−0.14	0.88	[−2.03, 1.75]

Abbreviations: CI, confidence interval; *GBA1*, glucocerebrosidase gene; PD, Parkinson’s disease. ^a^ Generalized Estimating Equations models were adjusted for age at diagnosis. The annual slope difference represents the interaction term between rasagiline use and time since diagnosis (years). ^b^ A negative value indicates a slower annual increase in motor scores (i.e., slower progression) among rasagiline users compared with non-users. The null hypothesis value is 0. ^c^ *p* values represent the statistical significance of the interaction term.

## Data Availability

Due to the presence of sensitive genetic and clinical information, individual participant data are not publicly deposited. However, de-identified participant data and the statistical analytic code (Python) will be made available upon reasonable request to the corresponding author. Access is subject to submission of a scientifically justified research proposal and required approval from the institutional review board. Hodaya Saragani, Mikhal E. Cohen, and Gilad Yahalom had full access to all study data and take responsibility for the integrity and accuracy of the data and analysis.
